# Impact assessment and cost-effectiveness of m-health application used by community health workers for maternal, newborn and child health care services in rural Uttar Pradesh, India: a study protocol

**DOI:** 10.3402/gha.v9.31473

**Published:** 2016-05-13

**Authors:** Shankar Prinja, Ruby Nimesh, Aditi Gupta, Pankaj Bahuguna, Jarnail Singh Thakur, Madhu Gupta, Tarundeep Singh

**Affiliations:** School of Public Health, Post Graduate Institute of Medical Education and Research, Chandigarh, India

**Keywords:** m-health, cost-effectiveness analysis, impact assessment, maternal and child health, community health worker, health services research

## Abstract

**Background:**

An m-health application has been developed and implemented with community health workers to improve their counseling in a rural area of India. The ultimate aim was to generate demand and improve utilization of key maternal, neonatal, and child health services. The present study aims to assess the impact and cost-effectiveness of this project.

**Methods/design:**

A pre–post quasi-experimental design with a control group will be used to undertake difference in differences analysis for assessing the impact of intervention. The Annual Health Survey (2011) will provide pre-intervention data, and a household survey will be carried out to provide post-intervention data.

Two community development blocks where the intervention was introduced will be treated as intervention blocks while two controls blocks are selected after matching with intervention blocks on three indicators: average number of antenatal care checkups, percentage of women receiving three or more antenatal checkups, and percentage of institutional deliveries. Two categories of beneficiaries will be interviewed in both areas: women with a child between 29 days and 6 months and women with a child between 12 and 23 months. Propensity score matched samples from intervention and control areas in pre–post periods will be analyzed using the difference in differences method to estimate the impact of intervention in utilization of key services.

Bottom-up costing methods will be used to assess the cost of implementing intervention. A decision model will estimate long-term effects of improved health services utilization on mortality, morbidity, and disability. Cost-effectiveness will be assessed in terms of incremental cost per disability-adjusted life year averted and cost per unit increase in composite service coverage in intervention versus control groups.

**Conclusions:**

The study will generate significant evidence on impact of the m-health intervention for maternal, neonatal, and child services and on the cost of scaling up m-health technology for accredited social health activists in India.

## Introduction

Globally, maternal and child mortality is declining, although the pace of decline is not sufficient to attain Millennium Development Goals (MDGs) 4 and 5 in almost 128 out of 137 developing countries (1). Significant progress has been made in India for maternal and child health survival. The maternal mortality rate has decreased from 487 to 190 maternal deaths per 100,000 live births between 1990 and 2013 (2). In terms of child mortality, since 1990, the under-five mortality rate has decreased from 126 to 53 deaths per 1,000 live births during the same period (2). Nearly two-thirds of maternal and child deaths in India are contributed by a few states including Assam, Uttar Pradesh (UP), Uttarakhand, Rajasthan, Madhya Pradesh, Chhattisgarh, Bihar, Jharkhand, and Odisha (3). Not surprisingly, the coverage of key maternal, neonatal and child health (MNCH) services in these states is very low. For instance in UP, the coverage of institutional deliveries, full antenatal care (ANC), and full immunization were 45.6%, 29.6%, 45.3%, respectively, in 2011–12 (4).

Since the introduction of the National Rural Health Mission (NRHM) in India in 2005, a number of interventions have been introduced to bolster the coverage of services such as institutional delivery, immunization, and antenatal and postnatal care (5). While NRHM focused on the supply side through strengthening of the government health care system, it also initiated steps to generate demand through community mobilization by creation of a new cadre of community health workers called accredited social health activists (ASHAs) (5). ASHAs act as village-level grass roots workers to generate demand for services. Harnessed properly, this vast pool of human resources can be a potent asset in the Indian government health system. An evaluation of ASHAs in 2011 found that although a 23-day training schedule has been developed by the Ministry of Health and Family Welfare, the quality of training needs to be strengthened in order to improve the performance of ASHAs (6).

To supplement community health worker training and retention of knowledge, mobile technology has been considered an effective and sustainable method in developing countries (7). With the widespread use of mobile phones in the rural areas of India, reliable health information can easily be made accessible even to the remotest areas (8). Thus, built-in tools with health messages in the mobile phones can be used by the community health workers as an aid for counseling pregnant women and nursing mothers. A study conducted in rural areas of Tamil Nadu in India to assess the feasibility of text messaging in delivering maternal and child health care showed that mobile health messages were well perceived and accepted in the community (9). Knowledge about the recommended minimum number of ANC visits was reported to increase from 10 to 37% after women received text messages for health promotion on mobile phones (9).

Use of technology in the health sector, especially in rural areas that have significant health workforce deficiencies, has been endorsed by the government of India at the highest level (10). In this context, the ReMiND (Reducing Maternal and Neonatal Deaths) project was introduced in two blocks of the Kaushambi district in UP. As part of this project, a mobile health (m-health) application that runs on open source CommCare software has been introduced as a job aid for ASHAs. This m-health platform tracks and supports clients for the ASHAs and provides individualized service and counseling. It replaces paper registers and flip charts with open source software that runs on inexpensive phones. During home visits, it aids ASHAs to register clients and provides real-time guidance through key counseling points, decision support, and simple referral algorithms.

Similar m-health interventions in other countries have proven beneficial. For example, in Afghanistan m-health resulted in 20% improvement in antenatal attendance and a 22.3% improvement in the number of women receiving skilled deliveries at a health facility (11). In terms of quality of counseling (i.e. whether a client receives complete and accurate information), a study in India showed that after a period of 4 months of use of the CommCare application, frontline workers had increased their knowledge retention of at least three danger signs across all key health categories, from 48% at baseline to 70% (12).

A study reviewed all the controlled trials of m-health interventions between 1990 and 2010 to determine the effectiveness of m-health technologies in improving health care service delivery (13). The results highlighted that generally consistent finding of modest benefits associated with the use of mobile interventions by health care providers for diagnosis and management, so these interventions may be appropriate for implementation. The study showed that although much research has assessed the effectiveness of m-health, high-quality evidence is still lacking in the literature (13).

Another systematic review on the economic evaluation of m-health has shown that there is a lack of concrete evidence to fully assess the economic impact of telemedicine, e-health, and m-health systems (14). Deficiencies in design of studies, such as lack of randomized control trials, small sample sizes, and absence of quality data and appropriate measures further limit the relevance of findings. Furthermore, evaluations of such interventions in low- and middle-income countries are almost negligible, and there is no evidence from India or other Southeast Asian countries (14).

We therefore propose to undertake an impact assessment and a cost-effectiveness study to assess the incremental cost per disability-adjusted life year (DALY) averted and incremental cost per unit increase in composite coverage indicator with use of an m-health intervention delivered through ASHA workers for strengthening MNCH services compared with routine MNCH service delivery in one of the districts of UP in northern India.

## Background

### Study setting

The study will be conducted in the Kaushambi district in UP. Out of the district's eight community development blocks, the m-health intervention has been implemented in two blocks: Mooratganj and Manjhanpur.

The Catholic Relief Services (CRS) with the help of a local NGO, Vatsalya, introduced the CommCare m-health application to 259 ASHAs in the two intervention blocks in 2012. The ASHAs in the ReMiND project were provided with basic Java-based mobile phones operating on open source CommCare software (15). Extensive trainings of ASHAs were done in both blocks on the use of these phones. The ReMiND application has tailored content and guides the ASHA through the course of a woman's pregnancy and newborn child care. ASHAs use the application for the following purposes:

1. To register each pregnant woman
2. To update her ANC record during home visits on the mobile application
3. To track her from pregnancy into the postpartum period
4. To track the health of the newborn
5. To track the status of routine immunization until 2 years of age
6. To provide appropriate counseling and support at each of these steps

**Fig. 1:**
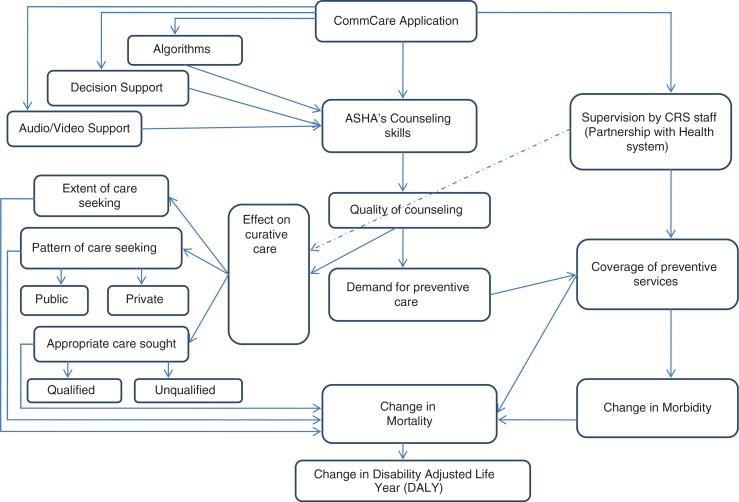
Conceptual frame work for impact of ReMiND project.

The phones contain locally relevant audio and visual prompts to help ASHAs navigate through the application. These recordings and pictures enrich the counseling experience of ASHAs during their home visits. The application uses the data entered about the pregnancy to guide ASHAs in providing timely and appropriate health information; it helps them to prioritize home visits; and it employs algorithms to assist in the early identification, treatment, and rapid referral to appropriate care of any danger signs among pregnant women or neonates (Fig. 1). While ASHAs have many tasks assigned to them, the key one is to identify pregnant women and visit them throughout the course of their pregnancy, seeing them through delivery in a safe facility and appropriate newborn care.

Data on services due and utilized by pregnant women, recorded by ASHAs through the m-health application, are pooled on a common server. The sector facilitators use the data to monitor all the ASHAs working in their area. These data are also shared with the health education officer at the primary health center level during monthly meetings. Thus, CRS works in coordination with the health system to monitor the performance of ASHAs using data generated by the m-health application (Fig. 1).

### Theory of change

The state of UP is one of the major contributors in total maternal and child deaths in India with low coverage of key MNCH services (3). Kaushambi is one of the 19 high focus districts in UP and exhibits some of the worst health statistics with maternal mortality ratio and infant mortality rate being 366 per 100,000 live births and 80 deaths per 1,000 live births, respectively (16, 17), which are higher in comparison with both national and state averages (4, 18). The district has a population of 1.2 million and a female literacy rate of 48.6% (19). An important reason for the lack of utilization of MNCH services is the reduced demand for services. This can be corroborated based on findings from Coverage Evaluation Survey which report that among pregnant women who do not seek ANC care, 55% do not consider it necessary. Similarly, 28% of women who deliver at home in UP did not consider it necessary to deliver in a health facility (20). This signifies a potential to increase utilization through demand generation.

One of the ways to generate demand is through quality counseling of women during pregnancy. Previous assessments of ASHA performance found them wanting in terms of their skills to counsel women effectively (21). Use of m-health with its associated audio–visual support is considered as a means for improving the quality of counseling services delivered through ASHAs. Improved knowledge about the need for services, together with an enabling environment with support from ASHAs, was likely to drive demand and hence utilization of MNCH services. Apart from this, continuous monitoring and supervision of ASHA performance through generation of real-time data on utilization of MNCH services as a result of the m-health application is also likely to contribute toward increased coverage of the preventive service. The m-health intervention includes five modules that could help ASHAs to track pregnant mothers in the village and ensure adequate ANC, postnatal care, newborn care, and immunizations through 2 years of age, which leads to the demand for MNCH services in the community finally leading to an increase in coverage of these services.

Increase in coverage of preventive services is likely to result in reduction of morbidities during pregnancy, child-birth, and neonatal period. Reduction in morbidity will in turn contribute to reduced mortality. The intervention is also likely to improve the care seeking during an episode of illness. Improved care seeking is likely to have an impact on mortality as a result of reduced case-fatality rates with treatment as compared with no treatment. Finally, the contact with public health services through the medium of ASHA workers may also change patterns of care seeking, that is, public versus private sector utilization of care. This is likely to affect the cost of health care services through reduction in out-of-pocket (OOP) expenditures and an increase in health system costs for provision of care. This is likely to have a bearing on the overall cost-effectiveness of intervention (Fig. 1) (22).

## Objectives

First, the primary objective of the present study is to assess the impact of the m-health application in the ReMiND project for ASHA workers on utilization of MNCH service coverage. Second, we aim to assess the cost-effectiveness of this m-health intervention in terms of incremental cost per DALY averted for delivering the intervention, as compared with routine services. Cost-effectiveness will also be assessed in terms of incremental cost per unit increase in composite service coverage indicator. Finally, we will assess the cost of scale-up for the m-health application, as it is implemented in the ReMiND project, at district and state level in India.

## Methodology

Impact assessment of the m-health intervention is provided in detail below.

### Overview of study design

The study has been approved by the Institute Ethics Committee of the Post Graduate Institute of Medical Education and Research, Chandigarh, India. Written informed consent will be obtained from all study participants. Administrative approval will be obtained for collection of relevant data.

We will use a quasi-experimental design comprising pre- and post-intervention observations with a control to assess the impact of the ReMiND project. Data from the Annual Household Survey (AHS) undertaken by the Registrar General of India in district Kaushambi in 2011 will be used for baseline or pre-intervention assessment (4). The AHS is the largest demographic survey in the world and covers two and a half times that of the Sample Registration System (India's most regular source of demographic statistics). The sampling design adopted for the AHS is a uni-stage stratified simple random sample without replacement, except in case of larger villages in rural areas (i.e. having a population greater than or equal to 2000 as per 2001 census), wherein a two-stage stratified sampling procedure has been applied. The details of the AHS survey are incorporated in the supplementary material.

We will undertake a cost-effectiveness analysis household (CEAHH) survey in 2015 in two intervention blocks and two control blocks from the Kaushambi district to assess the coverage of various MNCH services that represent the post-intervention status. To carry out the CEAHH survey, two structured interview schedules were developed after an extensive review of existing tools used in AHS, the National Family Health Survey, the District Level Household Survey, and the Coverage Evaluation Survey.

Together, the AHS and the CEAHH will be used to assess the impact of the ReMiND m-health intervention using a difference in differences (DID) analysis. To reduce selection bias, before DID is performed, propensity score matching (PSM) will be used to match the cases from the intervention blocks with those in the control group based on their socio-demographic characteristics, for the period between 2011 and 2015 (4). Further, subgroup analysis will be done to analyze the impact of intervention among various levels of underlying factors, such as the mother's education and the occupation of the head of household.

### Study area

The study area for the CEAHH survey will be two intervention and two control blocks out of a total of eight community development blocks in the Kaushambi district. The two control blocks will be selected based on matching for three health service utilization indicators: average number of ANC checkups, percentage of women receiving three or more ANC checkups and percentage of institutional deliveries.

### Sampling

The primary sampling unit (PSU) for the CEAHH survey will be a village. For selection of PSUs, a list of all villages from the two intervention and two control blocks will be prepared. From all villages in Kaushambi, 10% of each will be selected from the intervention area and the control area. This means that 69 PSUs each will be selected randomly from the intervention and control areas, using the probability proportional to size method.

In each PSU, a household enumeration will be undertaken to identify all mothers with a child in the age group of 29 days to 6 months, or between 12 and 23 months on the date of the survey. This list will serve as the sampling framework to randomly select the required number of mothers in each category for interview. The number of clients to be interviewed per PSU will again be selected by the probability proportional to size method and will be determined based on the relative size of the PSU.

The clients (mothers with a child in the 29 days to 6-month or 12- to 23-month age range) within each PSU will be selected using systematic random sampling. If more than one child in the respective age category is present in a given household (i.e. more than one child is between 29 days and 6 months or more than one child is between 12 and 23 months), then one will be randomly selected. If the family has one child in the 29 days- to 6-month range and one in the 12- to 23-month range, then both children would be part of the sampling frame and would have the same probability of being selected as would any other child in the sampling frame.

### Sample size

Two categories of individuals will be identified in each PSU: 1) women with one or more children aged 29 days to 6 months and 2) women with one or more children aged 12–23 months. The sample size for each category was estimated with respect to differences in coverage of two key MNCH services (institutional delivery and full immunization coverage) and neonatal and child morbidity rate for severe disease. For mothers with children in the younger age group, neonatal and child morbidity rates and institutional delivery coverage were used to determine sample size. For mothers with children in the older age group, the difference in rates of full immunization coverage was used to assess sample size.

We determined the sample size required to detect a 5% change in the coverage of MNCH services and neonatal and child morbidity rates, with a power of 80% and an alpha error of 5%. Baseline coverage of 15.1% and 30.8% for institutional delivery and full immunization coverage in control area, respectively, was assumed (18). Similarly, a neonatal morbidity rate of 18.9% for severe neonatal disease was assumed in the control area (4). With these parameters, sample sizes of 1,053 women with a child in the younger age group and 1,391 women with a child in the older age group from the intervention and control areas, respectively, were considered appropriate.

### Data collection

Basic socio-demographic data such as religion, caste, occupation, education, and wealth quantile will be collected from both categories of clients. Additionally, mothers of the younger group will be interviewed for utilization of ANC, institutional delivery, postnatal care, full immunization, neonatal & child morbidity, treatment sought and any hospitalization for the child. Information will be collected on the number of ANC visits, tetanus toxoid immunization, iron–folic acid supplementation, and type of health facilities used for ANC and institutional delivery. Data on OOP expenditures incurred at public or private health facilities for ANC and institutional delivery will be elicited. The mothers will also be questioned about any illness during the neonatal period, its symptoms, treatment sought, type of health facility, and OOP expenditures incurred. Similarly, mothers will be asked about any episode of hospitalization for the child, place of hospitalization, and OOP expenditures incurred.

Mothers of children in the older child group will be asked about immunizations received by the child. Data collection based on the immunization card would be preferable; however, mothers’ recall will be used in cases where an immunization card is unavailable in the home at the time of the interview. The use the mother's recall for eliciting information on immunization received by the child is a standard practice in developing and developed countries and is used in several household surveys, for example, Coverage Evaluation Survey, District Level Health Survey, National Family Health Survey, and Demographic Health Survey (20). Second, the mother's recall in the absence of immunization card for collecting the information on immunization status has been validated in a variety of different settings (23, 24). The mother will also be questioned for any illness episode the child may have experienced during last 15 days and any hospitalization in the last 365 days. For the latter two events, information on the place where treatment was sought and the OOP expenditures incurred will be sought.

Additionally, mothers in both categories will be interviewed in the intervention and control area about the extent and quality of services offered by the ASHAs during the antenatal and postnatal home visits. Specific questions will be posed to mothers in the intervention area to assess the usage of the mobile application and their satisfaction with this mobile-assisted counseling.

### Data analysis

DID will be the primary analysis and will be based on the AHS and CEAHH data from intervention and control areas. Propensity scores will be used to match the women of intervention area with control area based on their socio-demographic characteristics. This will help to reduce the selection bias by taking into account the variation caused by known confounders. The AHS dataset for analysis comprises a total of 450 women: 225 from the intervention areas and 225 from the control areas. The CEAHH data have 1,053 women with a child aged 1–6 months each in intervention and control area. Similarly, a total of 1,391 women with a child aged 12–23 months in intervention and control area are part of the CEAHH dataset.

(a) PSM: The non-random choice of the two intervention blocks could result in selection bias. In order to minimize this bias, the PSM method will be used to control for demand-side characteristics that could influence utilization of various MNCH services. The PSM approach attempts to render the effects of different observed covariates X on participation as a single propensity score or index defined by

P(X)=Pr(T=1∣X).

The idea is to find, from a large group of control area women, those who are observationally similar to the intervention area women in terms of characteristics that are unaffected by the m-health intervention but that influence the utilization of key MNCH services (25). Then, each woman in the intervention arm will be matched with a woman in the control arm who is observationally similar (e.g. on socio-economic characteristics like literacy rate, caste, occupation of the mother, and age). The PSM estimator for the treatment effect on the treated can be specified as the mean difference of Y (i.e. institutional delivery or full immunization, or any other key indicator under consideration) over the usual support, weighting the comparison units by the propensity score distribution of eligible women in the respective categories. A typical cross-section estimator can be specified as follows:

$$TOT_{PSM}=E_{P(x)|T=1}\{E[Y^{T}|T=1,P(X)]-E[Y^{C}|T=0,P(X)]\}.$$

(b) DID: This method essentially compares intervention and control groups in terms of outcome changes over time relative to the outcomes observed for a pre-intervention baseline. That is, given a two-period setting where *t*=0 before the program and *t*=1 after program implementation, letting $Y_{t}^{T}$ and $Y_{t}^{C}$ be the respective outcomes for a program beneficiary and non-treated units in time *t*, the DID method will estimate the average program impact as follows:

$$DID=E(Y_{1}^{T}-Y_{0}^{T}|T_{1}=1)-E(Y_{1}^{C}-Y_{0}^{C}|T_{1}=0)$$

In equation (1), *T*_1_=1 denotes treatment or the presence of the program at *t*=1, which is evaluated from CEAHH data, whereas *T*_1_=0 denotes absence of the program, which is evaluated using AHS data. Outcomes in the form of coverage of key MNCH services such as ANC, immunization and institutional deliveries will be assessed (25). Along with the above techniques, for each primary and secondary outcome, the estimated effect size and confidence intervals will be estimated to indicate the levels of precision.

### Quality control

Data quality will be assessed during the survey as adherence to the sampling plan, correctness, completeness and accuracy of data. With these aspects of quality in mind, a team of supervisors will monitor the sampling techniques followed by field investigators while collecting the data. During the quality visits, 10% of the data collected by each investigator will be collected again by the supervisor and further analyzed to check for discrepancies. If a wide range of discrepancies within or across investigators or items is found, a standard set of procedures, for example, validation or repeat sampling procedures, will be followed to resolve the same. Missing data will be imputed with the standard regression imputation technique.

#### Costing and cost-effectiveness assessment

First, costing will be undertaken from a societal perspective. Data on OOP expenditures for accessing MNCH services such as ANC, institutional delivery and curative care (including outpatient consultation and hospitalization) during the neonatal and infancy period will be collected during the CEAHH survey. A bottom-up costing method will be used to collect data on health system costs of implementing the m-health application as in the ReMiND project. This will comprise data on start-up costs such as planning meetings, development of software, and training of ASHAs. The initial start-up costs (such as trainings and software development) will be considered as capital costs and will be annualized based on the expected life of the product produced. Second, these will be apportioned to the two blocks of UP, assuming a scenario that the application can be scaled up in all of UP without further adaptation. Cost of implementation of the program will be assessed in terms of resources of implementing partners such as CRS and Vatsalya, which implemented the program in the intervention area. These resources include staff salaries, equipment, building and space, drugs and consumables, and other overheads. Health system costs for delivering services through ASHAs will be assessed by collecting data on performance-based incentives paid to the ASHAs. For the purpose of cost scale-up analysis, only those start-up costs will be considered that are likely to be incurred by the health system as part of scale-up – for example, adaptation of the software in the new state, trainings, and any manuals to be developed.

Other health system costs that are incurred for delivery of preventive and curative MNCH services will be drawn from published studies (26–28). Together with data on health service utilization and OOP expenditures (as obtained from the CEAHH survey), epidemiological parameters (as obtained from a review of literature) and data on health system costs collected from the present study, overall cost of delivering MNCH health services will be estimated. This will also be used to generate the estimate for standardized cost of delivering MNCH services (preventive and curative) at the district level, with and without the m-health application.

**Fig. 2:**
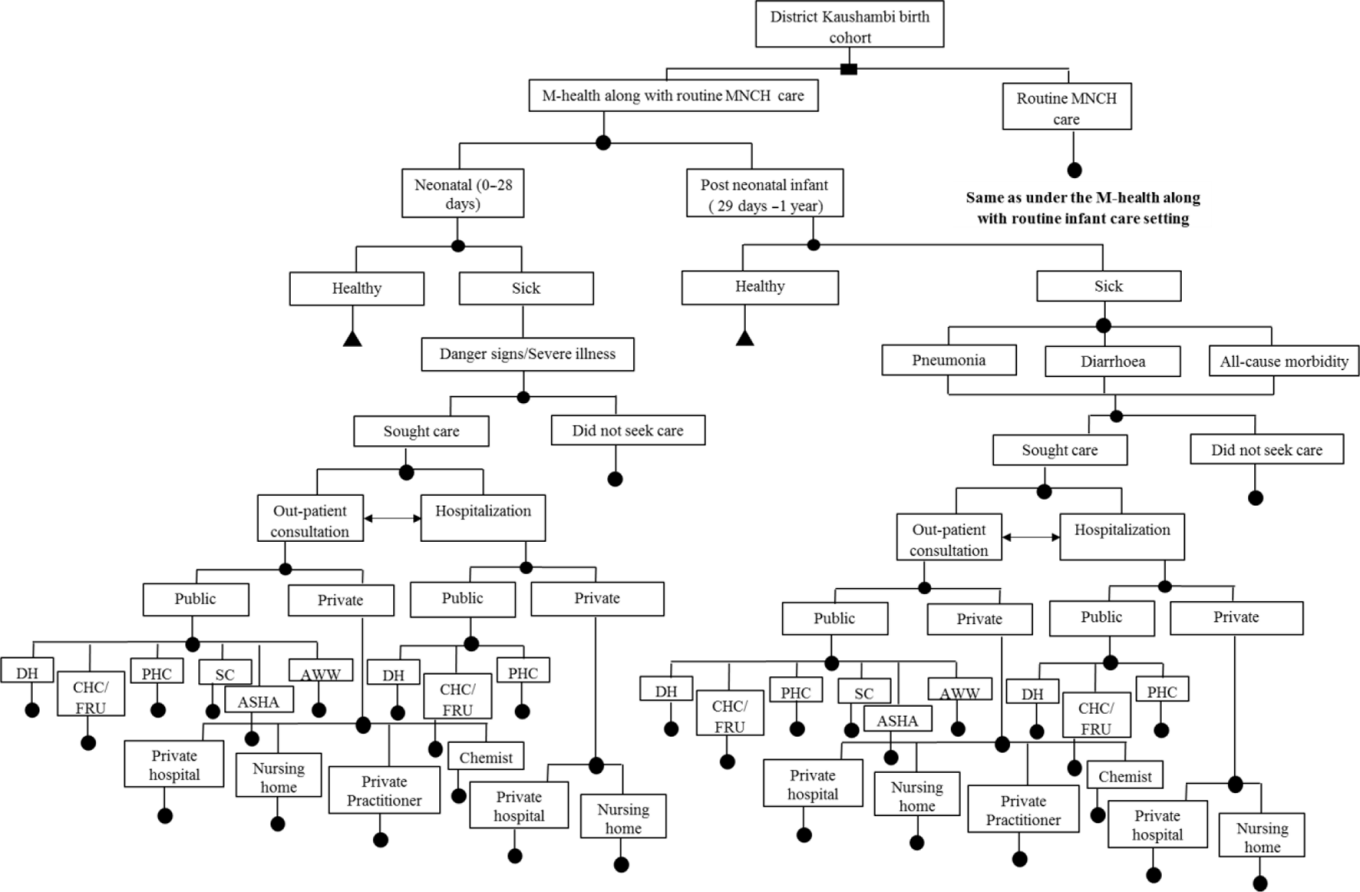
Decision model for cost-effectiveness study of m-health application for ASHA workers as part of ReMind project. Note: DH=district hospital, CHC=community health center, FRU=first referral unit, PHC=primary health center, SC=sub-center, ASHA=accredited social health activist, AWW=Anganwadi worker, MNCH=maternal neonatal and child health. Cycle repeated for 15 birth cohorts. Source: This model is adapted from a similar figure in Ref. (29).

**Fig. 3:**
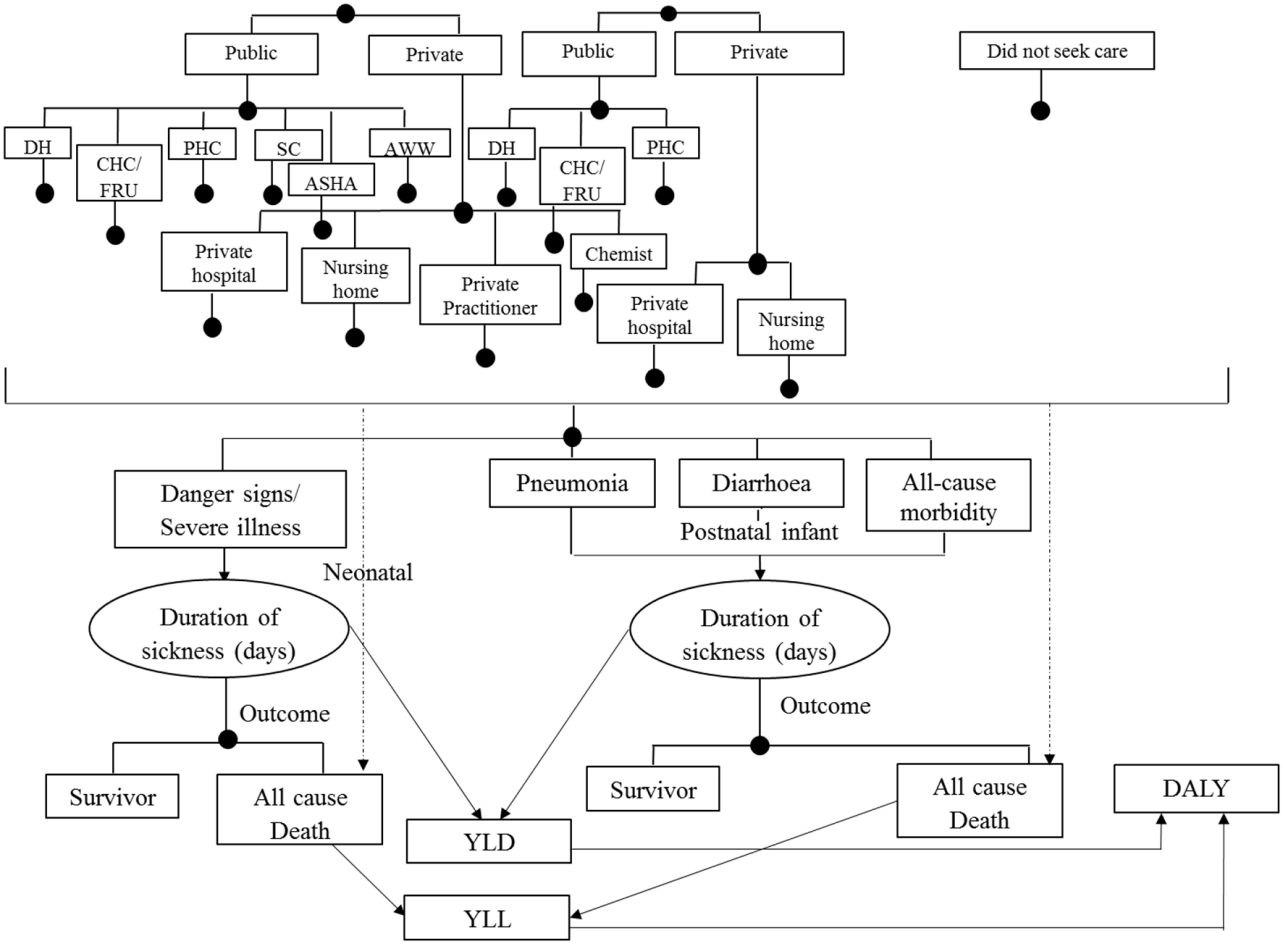
Outcome model for cost-effectiveness study of m-health application for ASHA workers as part of the ReMind project. Note: In continuation to the model 1, outcome model describes the probable scenarios after a neonatal or post-neonatal infant had been treated or not (irrespective of type of health facility). YLD=years of life lived with disability, YLL=years of life lost due to premature mortality, DALY=disability-adjusted life years. Source: This model is adapted from a similar figure in Ref. (29).

**Fig. 4:**
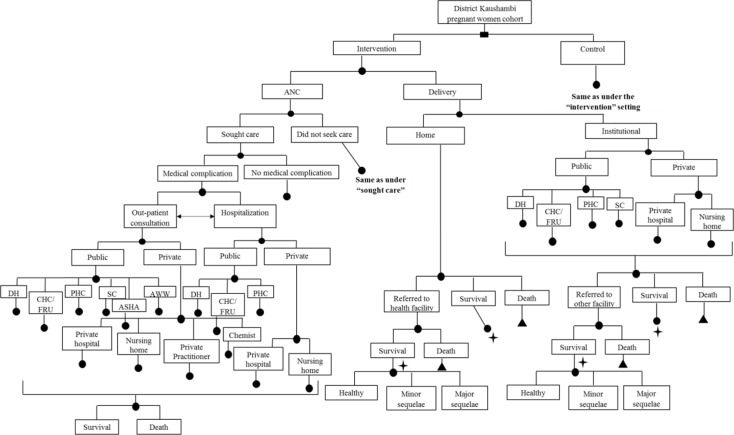
Decision model for cost-effectiveness (maternal health) study of m-health application for ASHA workers as part of ReMind project.

An existing model to evaluate the impact of IMNCI would be adapted and used to undertake the cost-effectiveness analysis (29). A decision model will be developed using an MS-Excel spreadsheet to estimate the incremental cost-effectiveness of implementing the m-health application for ASHAs as used in the ReMiND project (Figs. 2–4). A time horizon of 10 years starting from base year 2011 was considered appropriate to cover all costs and effects comprehensively. Ideally, period of time horizon should be such that it covers all important costs and consequences as a result of the intervention. In the case of ReMiND project, several reasons justify the time horizon of 10 years. First, the m-health software is unlikely to change in this period as the broad nature of services will remain same. Second, based on expert opinion even if the software has to be edited based on revisions in the program package, such changes are unlikely to have any major cost implications. Third, the health system programs also have a shelf life, and it has been observed that incremental changes in the program design are usually made over an approximate 10-year period. However, majority of the diseases show their effects in the childhood period, that is, 0–5 years of age and some other disease may take even longer (10 years) to show their effects. In view of both situations, we believe that a 10-year time horizon will be appropriate to account for all the costs and benefits that accrue as a result of the intervention.

Costs and benefits will be analyzed from both a health system and a societal perspective. Benefits on health status will be measured in terms of illness episodes averted, child deaths prevented, maternal deaths prevented, life years gained and DALYs averted. Evaluation of health benefits will be undertaken by modeling the improvements in service utilization and reduction in illnesses during the neonatal period and infancy, as well as reduction in maternal and infant mortality and disability. Finally, reduction in DALY as a result of implementation of the m-health application will be estimated. The effects on maternal and infant mortality will be modeled for the following intermediate consequences: improvement in ANC and institutional delivery; reduction in neonatal and infant morbidity, predominantly pneumonia and diarrhea; and improvements in care seeking for sick newborns. The existing evidence based on the LiST model and pertaining to investigation of improvements in MNCH-related preventive services will be reviewed to assess these relationships. This will build on the existing evidence that is used for modeling the effect of improvements in these preventive services, as well as care seeking in the LiST (Lives Saved Tool) model (30). Benefit will also be measured in terms of improvement in service coverage. A composite service indicator will be developed to assess the change in service coverage.

A composite coverage measure will be computed by taking the arithmetic mean of output indicators, viz., institutional delivery, full ANC, and full immunization coverage. The data on each of the input indicators is on a ratio scale. However, these are non-comparable as they measure different dimensions of health services (e.g. maternal health and child health). Therefore, geometric means will be used for aggregating. Because all the input indicators are in the form of percentage ranging from 0 to 100, there is no need for scaling. Weights will also be assigned, which will be derived through expert opinions and published literature. The nature of current data available does not support determining of weights with multivariate statistical methods such as principal component analysis or regression analysis. Both costs and benefits will be discounted at 3% to account for time preference of cost and utility (31). We will estimate the standardized unit cost, from a health system and a societal perspective, for implementing MNCH services in the scenario of routine services plus m-health application versus routine services alone. We will report our findings as incremental cost of implementing per DALY averted as compared with routine care services alone.

## Process evaluation

The data would be collected from the ASHAs in intervention and control areas to assess their knowledge, beliefs and attitudes about the acceptability of the application based on self-administered ASHA knowledge assessment tool. A self-administered and semi-structured questionnaire was developed to interview the ASHAs, which was based on the ASHA training modules under National Health Mission and an earlier study undertaken for evaluation of ASHA performance (32, 33). The draft tool was subsequently edited based on the inputs received from the state head of ASHAs division in UP, District authorities–Chief Medical Officer, Deputy Chief Medical Officer, District Program Manager, and District Community Process Manager. The tool consisted of both close-ended and open-ended questions regarding the use of the m-health intervention which involved four sections, viz., general information, assessment of ASHA's activities, assessment of ASHA's knowledge about maternal and child health, and perception about m-health application. Also, the tool involved the individual views of participants regarding the merits and demerits of using m-health intervention. The evaluation will be conducted in all four blocks. Within each intervention and control blocks, 10% of the ASHAs from all primary health centers (PHC) will be selected randomly for the present evaluation.

We will also monitor the implementation of any other intervention or change in the intensity of existing interventions in the study area during the period of evaluation, especially to determine whether the additional or existing interventions are implemented differentially between the intervention and control area.

## Discussion

Health care interventions have been traditionally evaluated using randomized controlled trials, which are considered as the best form of evidence. However, allocation of usual health system interventions in routine programmatic settings is not randomly allocated, which poses special challenge to researchers for evaluation (34). As a result, several solutions have been put forth, such as using district as the focus of evaluation, employing multiple data sources, and careful selection of control population (35). In the present study, we have adopted a pre–post quasi-experimental design along with a control group. Control area was selected based on matching for key variables. Multiple data sources, for example, AHS 2011 and CEAHH 2015, are being used to assess the impact on coverage of services. Different analytical techniques are incorporated to control for confounding factors and biases, such as PSM and DID analysis.

The present study is being undertaken to assess the impact and cost-effectiveness of an m-health application used by village-level ASHAs for improving MNCH services. To date, per our knowledge there is no cost-effectiveness evaluation of an m-health application delivered through community health workers for maternal and child health care from India or any other Southeast Asian country (13). More than 850,000 ASHAs have been recruited under the National Health Mission – India's flagship health program – with the primary purpose of being a social agent for generating demand for health care services (36). Moreover, a recent report clearly brought out the deficiencies in the skills of ASHAs to effectively counsel pregnant women and mothers during the postnatal period. This has a bearing on the utilization of services and on the knowledge of the beneficiaries. A number of strategies are being considered for improving the skills of these ASHAs. Besides the routine training programs, use of such mobile applications is being considered as one important strategy. Moreover, mobile applications have received a boost at the highest political level, with the government of India promoting the application of technology-based solutions in delivery of health care.

In view of these health system and policy debates, the present evaluation holds immense significance to provide evidence from an economic viewpoint on whether such m-health interventions should be scaled up. Hence, it is a comprehensive study that evaluates the intervention thoroughly in terms of its process evaluation, impact, cost, and cost-effectiveness.

It has been argued by others that researchers should consider enough period of implementation for their evaluation to begin, such that intervention has sufficient time to demonstrate its impact. Generally, it has been seen that the incremental community-based interventions in the field of maternal and child health bring out a change in about 2–4 years period, especially in areas with the low coverage of health indicators (37–40). Similarly, a review of effectiveness of m-health services includes several studies that had used a similar time frame as proposed in our study (13). Second, in areas with low coverage of services, there is greater likelihood of early adopters that will manifest in a change in shorter term compared with area where the coverage of services is high (37). Since UP state in general is low coverage, and district Kaushambi is even a poor performing district within UP state, we believe that the intervention is likely to show the impact in the coverage of services within time period being considered in the study.

We note certain limitations of the present study. Design of health program evaluations has been dominated traditionally by experimental approaches used in medicine, in which specific individuals or clusters of people are allocated randomly to receive an intervention whereas others do not. Studies tend to be undertaken in controlled environments in which the influence of external factors is minimized or eliminated. Such randomized controlled trials are considered to provide the highest quality of evidence to evaluate an intervention or program. However, real-world health system interventions are far from controlled experiments (41). Moreover, the policy makers are usually interested in rolling out the program in the entire geographic area, with little chance for researchers to design studies that have adequate power to establish cause–effect relationships (35).

In the context of the present study, the intervention is to be rolled out in two blocks in the district. This offers distinct benefits for evaluating the impact. However, the choice of the two intervention blocks was not random, and this selection bias can limit the causal attribution if inherent differences in the two intervention blocks versus the remaining six control blocks of the district influence the outcome. However, we will attempt to account for this in our analysis, where the demand-side factors at household level will be controlled using PSM, and the supply-side health system factors other than m-health application are controlled using the DID estimator.

Second, the intervention involves use of mobile phones to deliver health messages and counseling. Given the nature of the intervention, it is not possible to blind the participants. Also since specific questions pertaining to the use of intervention are included in the structured questionnaire on delivery of intervention, the investigators who assess outcomes also cannot be blinded to the intervention.

Third, we aim to assess provision of home-based postnatal care through ASHAs, morbidity and care seeking during the neonatal and infancy periods. We note that these were not the primary objective of ReMiND, as its m-health application was limited to being a job aid for ANC, institutional delivery, and immunization. However, since the m-health application might affect the performance of ASHAs and their contact with the women during pregnancy, it is likely to have spin-off effects during the postnatal period. Whatever differences accrue for neonatal care seeking, postnatal care, and so on, the same differences will be modeled as health gains in the form of DALY averted. Moreover, the baseline survey elicited data on provision and quality of ASHA visits during the postnatal period, neonatal morbidity and care seeking. Hence, it is feasible to assess impact, if any.

There are also some limitations in the way DALYs and composite coverage indicators are defined. It is well known that the maternal and child health services have many non-health benefits. For example, healthy children have better school performance, and non-anemic women have better productivity. However, to capture such benefits is outside the scope of the present study. Similarly, the creation of composite indicators is also methodologically debated in terms of the weights allocated to each input indicator. In order to address this uncertainty, we will undertake a sensitivity analysis to account for the variability introduced with and without weighting. However, as mentioned previously, the nature of current data (coverage available for two units only; i.e. intervention and control) does not support determination of weights using multivariate statistical methods such as principal component analysis or regression analysis.

Overall, the present study will generate estimates on incremental cost per DALY averted with the m-health application intervention in comparison with routine services. The study will also be able to generate evidence on the cost of scale-up for such an intervention, which will address important fiscal feasibility issues. Together, the two will help the government of UP and the government of India to consider replication of such an intervention in the entire state or country. India is a diverse nation with considerable heterogeneity in terms of baseline levels of service utilization, organization of the health care delivery system and its quality. As a result, several factors will need to be considered to generalize the findings of the study in India. These include the characteristics of the study population and of the intervention, the setting of the study, and other contextual issues, all of which will be interpreted in the context of the current evidence available in the literature.

## Supplementary Material

Impact assessment and cost-effectiveness of m-health application used by community health workers for maternal, newborn and child health care services in rural Uttar Pradesh, India: a study protocol

## Authors’ information

SP is an associate professor of health economics who has special interest in undertaking economic evaluation of health care programs and interventions and impact evaluations of public policies and health programs. PB and RR are statisticians who have extensive experience in developing study designs. Additionally, PB has contributed to several economic evaluations for developing decision-analytic models. AG is a public health specialist. JST and MG are community physicians with special interest in maternal and child health care, and TS is a community physician with interest in health informatics.

## Paper context

M-health applications are being used to improve the effectiveness and efficiency of interventions delivered through community health workers. Despite some evidences on impact of these technology based interventions, there is significant gap in evidences on cost-effectiveness. Our study, which is being undertaken in high burden and resource constrained setting in India, proposes to assess the impact and cost-effectiveness of an m-health intervention delivered through community health workers to improve maternal and newborn survival.
