# Supplementary material for: Impact assessment and cost-effectiveness of m-health application used by community health workers for maternal, newborn and child health care services in rural Uttar Pradesh, India: a study protocol
Source: Glob Health Action. 2016 May 13;9:10.3402/gha.v9.31473. doi: 10.3402/gha.v9.31473 (PMC4870358; doi:10.3402/gha.v9.31473)
Supplement: Impact assessment and cost-effectiveness of m-health application used by community health workers for maternal, newborn and child health care services in rural Uttar Pradesh, India: a study protocol [file GHA-9-31473-s001.docx]

Supplementary information.

**Title: Impact assessment and cost effectiveness of m-health application used by community health workers for maternal, newborn and child health care services in rural Uttar Pradesh, India: study protocol**

Running Title- Cost effectiveness of an m-health intervention-a protocol

Authors: Shankar Prinja, Ruby Nimesh, Aditi Gupta, Pankaj Bahuguna, Jarnail Singh Thakur, Madhu Gupta, Tarundeep Singh

**Methodology of AHS Survey**

The AHS is the largest demographic survey in the world and covers two and a half times that of the Sample Registration System (India’s most regular source of demographic statistics). Decentralized district-based health planning is essential in India because of the large inter-district variations. In the absence of vital data at the district level, the State level estimates are being used for formulating district level plans as well as setting the milestones thereof. In the process, the hotspots (districts requiring special attention) very often get masked by the State average. This statistical fallacy compounds the problems of the districts acutely, more so in the health sector. At present, none of the Surveys provides estimates of core vital indicators on fertility and mortality at district level. The District Level Household Survey conducted with periodicity of five years mainly focuses on indicators pertaining to maternal health and child welfare programmes. There has, therefore, been a surge in demand from various quarters, in recent years, to generate timely and reliable statistics at the district level for informed decision making in the health sector.

The Annual Health Survey (AHS) was conceived during a meeting of the National Commission of Population held in 2005 under the Chairmanship of the Prime Minister wherein it was decided that “there should be an Annual Health Survey of all districts which could be published / monitored and compared against benchmarks”. The objective was to monitor the performance and outcome of various health interventions of the Government including those under National Rural Health Mission (NRHM) at closer intervals through these benchmark indicators. The AHS has been made an integral part of the NRHM, Ministry of Health & Family Welfare. The responsibility of the project has been entrusted to the Office of the Registrar General, India on behalf of the Ministry of Health & Family Welfare in view its expertise in handling the Sample Registration System, one of the largest demographic surveys in the world.

Realizing the need for preparing a comprehensive district health profile on key parameters based on a community set up, the AHS was designed to yield benchmarks of core vital and health indicators at the district level; prevalence of disabilities, injuries, acute and chronic illness and access to health care for identified morbidities; and access to maternal, child health and family planning services. By virtue of being a panel survey, it has the unique ability to map the rate of change in these indicators on a yearly basis. AHS would, thus, enable better capturing of the health seeking behaviour of the public as compared to other periodic cross-sectional surveys, and also help needed corrections in the strategies.

The sample size at the district level has been derived taking Infant Mortality Rate as the decisive indicator and host of other practical issues related to execution of the survey. Keeping in view, the mammoth size of the sample in mind, they initially confined our selection to the 284 districts (as per 2001 Census) in the eight Empowered Action Group States (Bihar, Jharkhand, UP, Uttarakhand, Madhya Pradesh, Chhattisgarh, Orissa and Rajasthan) and Assam for a three-year period starting from 2010 to 2011. These 9 high focus States with relatively high fertility and mortality account for about 48 percent of the total population in the country. A representative sample of 20,694 statistically selected Primary Sample Units (PSUs - Census Enumeration Blocks in case of urban areas and villages or a segment thereof in case of larger villages in rural areas) based on 2001 Census has been drawn from these AHS States which would cover about 18 million population and 3.6 million households each year. However, during the Base-line Survey, a total of 20.1 ii million population and 4.14 million households and during the first updation survey, 20.61 million population and 4.28 million households have actually been covered. Despite being restricted to 9 States, the AHS is the largest demographic survey in the world and covers two and a half times that of the Sample Registration System.

The sampling design adopted for the AHS is a uni-stage stratified simple random sample without replacement, except in case of larger villages in rural areas (i.e., having a population greater than or equal to 2000 as per 2001 census), wherein a two-stage stratified sampling procedure has been applied. The sample units are census enumeration blocks in urban areas and villages in rural areas. In rural areas, the villages have been divided into two strata, where Stratum I comprises villages with a population less than 2000 and Stratum II contains villages with a population 2000 or more. The number of sample villages in each district was allocated between the two strata proportionally to their size (population). The sample villages within each substratum were selected by simple random sampling without replacement. Further, the process of selection ensured equal representation across three sub-strata (which were based on the female literacy rate) in both rural and urban areas of a district, and rendered the sample design as self-weighting(4).

One of the essential prerequisites for the commencement of the survey was to uniquely identify the sample units on ground. This was done in all the sample units across the nine AHS States by the regular staff of ORGI. The work involved firming up of the boundary of the selected villages / Enumeration Blocks; resorting to segmentation in case of villages exceeding the population 2000, random selection of segment thereof and drawing of appropriate notional maps of the sample units to serve as the base map for the survey work.

The first updation survey in all the nine AHS States was carried out during October 2011 to April 2012 and four Schedules, in all, were administered. These are: (i) House-listing Schedule, (ii) Household Schedule, (iii) Woman Schedule and (iv) Mortality Schedule. In the House-listing Schedule, the mapping, listing and some key particulars like type and ownership details collected in the baseline survey in 2010-11 were updated for the existing houses and households and recorded afresh for the new houses and households in the first updation round.

During the first updation survey, all the Usual Residents as on 01.01.2011 were listed in the Household Schedule wherein the information on a few back ground characteristics viz. Name, Sex, Identification Code, Date of Birth and Date at first Marriage were copied from the baseline Household Schedule for the Usual Residents of baseline survey and other characteristics like Relationship to Head, Age, Religion, Social Group, Marital Status, Education and Occupation/Activity Status were captured afresh for them . For the new Usual Residents as on 01.01.2011, all the details were captured afresh. Besides, information in respect of Disability, morbidity (Injuries, Acute Illness, and Chronic Illness) and access to health insurance is also captured for all the usual residents as on 01.01.2011.

Woman Schedule comprised two sections. Section-I was administered to all Ever Married Women (EMW) aged 15-49 years and information relating to the outcome of pregnancy(s) (live birth/still birth/abortion); birth history; type of medical attention at delivery; details of maternal iv health care(ante-natal/natal/post-natal); immunization of children; breast feeding practices including supplements; occurrence of child diseases (Pneumonia, Diarrhoea and fever); registration of births, etc. taken place during the reference period( i.e. 01.01.2010 to 31.12.2010) was collected. Section II focused on information on pregnancy; use, sources and practices of family planning methods; details relating to future use of contraceptives and unmet need; awareness about RTI/STI, HIV/AIDS, administration of HAF/ORT/ORS during diarrhoea and danger signs of ARI/Pneumonia; and these details were collected from all Currently Married Women aged 15-49 years. Information relating the Ever Married Women (EMW) like conception details, usage of NPT kit, registration of pregnancy, health problems and subsequent treatments during ante-natal/natal/post-natal period, cost incurred by the woman during delivery etc. is also collected during the first updation survey.

Through the Mortality Schedule, details relating to death occurred to usual residents of sample household during the reference period (01.01.2010 to 31.12.2010) were captured and it included information on name & sex of deceased, date of death, age at death, registration of death and source of medical attention received before death. For infant deaths, a question on symptoms preceding death was also probed. In case of deaths associated with pregnancy, information on a variety of questions on factors leading/contributing to death, symptoms preceding death, time between onset of complications and death, etc. were asked to yield data on various determinants of maternal mortality.

In addition to the multilayer supervision mechanism adopted by the Survey Agencies, regular inspections were carried out by the officers/officials of respective DCOs and those from ORGI headquarters to ensure the data quality. The inspections were a judicious mix of concurrent as well as post survey audit. Over and above, a component of Third Party Audit was included to verify and authenticate the surveyed data through an independent mechanism. The Third Party Audit work was carried out in 20 randomly selected AHS units in each of the districts covering every household thereof by following a standard protocol prescribed by ORGI. Truncated versions of Household, Women and Mortality Schedules were filled in afresh by the field staff of the Third Party Audit Agencies. The findings in respect of key indicators were matched and re-survey was undertaken by the Survey Agencies in units where the variation was outside the permissible limit. The third party audit also helped in netting of missed vital events, particularly rarer ones like infant and maternal deaths.

In view of the large volume of data collected under AHS and significant time required for validation and processing, dissemination of AHS results is done in two phases. The first set of data of the first updation survey was released in May 2013 in the form of State-wise bulletins, which contained the district level data on crude birth rate, crude death rate, natural growth rate, infant mortality rate, neo-natal and post neo-natal mortality rates, under 5 mortality rate, sex ratio at birth, sex ratio (0-4 years) and overall sex ratio. In addition, the Maternal Mortality Ratio (MMR), Maternal Mortality Rate and life time risk were released for a group of districts. In order to facilitate direct intervention, the maternal mortality indicators were combined and released for a group of districts on the basis of existing administrative divisions in the respective AHS States.

Under the present phase of dissemination, data on host of other important parameters covered in AHS under Household and Woman Schedules are being released in the form of v State and District Level Factsheets. Though the sample size has been calculated for the district as a whole, the rural and urban estimates at the district level have also been published as byproduct. Users are advised to keep the above fact into consideration while using the rural / urban estimates of a district. In order to ward off unusual sampling fluctuations, the urban estimates have not been published in respect of some indicators for the districts where the number of urban sample units was less than six. To begin with, number of PSUs, Households, Population, Ever Married Women, Currently Married Women, Children aged 12-23 months (as on date of survey) covered in the sample of each district and the State along with their rural-urban breakup have been given to provide the users requisite insight on the metadata. The indicators contained in the AHS Bulletin have also been reproduced in these Factsheets so that the users may have access to complete set of indicators at one place. However, they may refer to the AHS Bulletin for details on metadata.
